# The Potential of Vitamin D-Regulated Intracellular Signaling Pathways as Targets for Myeloid Leukemia Therapy

**DOI:** 10.3390/jcm4040504

**Published:** 2015-03-25

**Authors:** Elzbieta Gocek, George P. Studzinski

**Affiliations:** 1 Faculty of Biotechnology, University of Wroclaw, Joliot-Curie 14a, Wroclaw 50-383, Poland; E-Mail: elzbieta.gocek@uni.wroc.pl; 2 Department of Pathology, New Jersey Medical School, Rutgers, The State University of New Jersey, 185 South Orange Ave., Newark, NJ 17101, USA

**Keywords:** acute myeloid leukemia, targeted therapy, differentiation, 1,25-dihydroxyvitamin D_3_, mitogen-activated kinases

## Abstract

The current standard regimens for the treatment of acute myeloid leukemia (AML) are curative in less than half of patients; therefore, there is a great need for innovative new approaches to this problem. One approach is to target new treatments to the pathways that are instrumental to cell growth and survival with drugs that are less harmful to normal cells than to neoplastic cells. In this review, we focus on the MAPK family of signaling pathways and those that are known to, or potentially can, interact with MAPKs, such as PI3K/AKT/FOXO and JAK/STAT. We exemplify the recent studies in this field with specific relevance to vitamin D and its derivatives, since they have featured prominently in recent scientific literature as having anti-cancer properties. Since microRNAs also are known to be regulated by activated vitamin D, this is also briefly discussed here, as are the implications of the emerging acquisition of transcriptosome data and potentiation of the biological effects of vitamin D by other compounds. While there are ongoing clinical trials of various compounds that affect signaling pathways, more studies are needed to establish the clinical utility of vitamin D in the treatment of cancer.

## 1. Introduction

Cytotoxic therapy can be quite successful in the control of the growth and dissemination of many human malignant diseases, but the established treatment regimens appear to have reached a plateau in their potential for improvement. Therefore, encouraged by the success of Imatinib mesylate, also known as Gleevec, in producing long-lasting remissions of CML by targeting the fusion gene Bcr-Abl with tyrosine kinase activity [1,2,3,4] and of ATRA, which targets a fusion TF PML-RARα in acute promyelocytic subtype M3 of AML (APL) [5,6,7,8,9,10], the search is on for similar targeting of other neoplastic diseases. Derivatives of vitamin D (VDD) have been suggested to have anti-neoplastic properties, but the translation of the results of epidemiological and laboratory studies to the clinic has so far not been successful [11,12,13,14]. In this review, we discuss the background for seeking molecular targets related to signaling pathways that current knowledge suggests have the potential for the exploration of their clinical usefulness in subtypes of AML other than CML and APL. While several excellent reviews have been published recently that overlap with this one [15,16,17,18], our aim is to update this knowledge, as well as to focus on several selected aspects of the vitamin D and human leukemia field that we feel deserve additional emphasis.

## 2. Signaling Pathways Studied in Hematopoietic and Myeloid Cells

AML is a predominant acute leukemia among adults and constitutes a very heterogeneous group of blood and bone marrow neoplasms [19,20,21]. AML is an aggressive disease characterized by over 20% of myeloblasts circulating in the blood or/and bone marrow [20,22,23,24]. Blast cells are characterized by inhibited differentiation, as well as increased proliferation. Moreover, AML have specific cytogenetic and molecular abnormalities [25]. There are more than two hundred described chromosomal aberrations in leukemic cells of patients with AML [26,27], but also a large group of AMLs without detectable cytogenetic abnormalities [20,28]. 

Despite significant improvements in chemotherapeutic regimens, poor responsiveness and relapse are still problems in a significant number of patients diagnosed with AML. The clinical outcome with chemotherapy alone is still abysmal for many myeloid leukemia patients, so the development of precision therapy, also called “targeted” therapy, for AML patients based on the molecular features remains an essential aim. Therefore, there is a great need for new therapies with better tolerability and effectiveness than the current treatments. As mentioned above, APL was the first hematological malignancy in which targeted therapy with ATRA has been successfully introduced into clinical practice and induces cell differentiation and death of blast cells [29,30,31]. Another compound capable of inducing differentiation of AML cells is 1,25-dihydroxyvitamin D_3_ (1,25D), which induces monocyte/macrophage-like differentiation and cell cycle arrest [32,33,34,35,36]. The importance of understanding the signaling pathways disturbed in AML cells may improve current treatments and may supplement the conventional therapeutic regimens.

### 2.1. MAP Kinase Signaling

**Figure 1:**
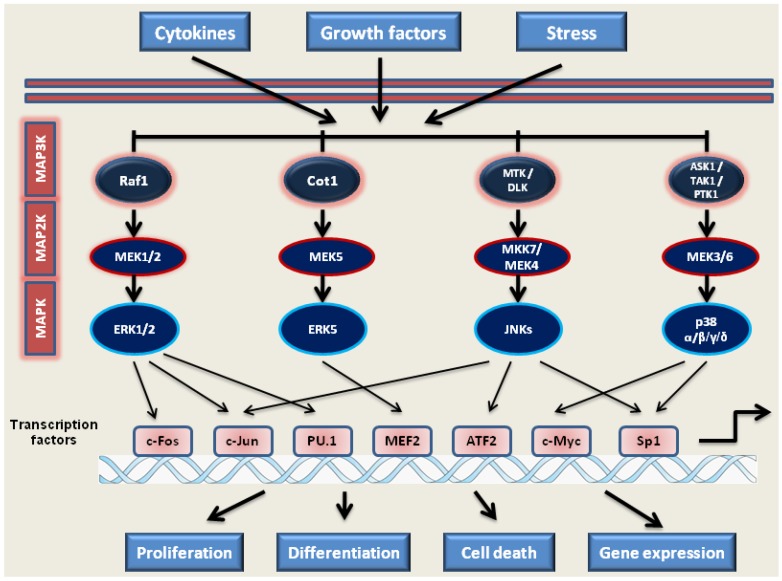
MAPK signaling pathways.

The MAPKs constitute a family of serine-threonine kinases regulating the proliferation and differentiation of normal and malignant hematopoietic cells [37,38]. MAPKs signal by four main cascades: the ERK1/2, the JNKs, the p38 kinases and ERK5 kinase [37,39] (Figure 1). There are multiple interactions between these pathways, including cooperation and cross-talk between various components, in order to transmit specific signals to the cell [40,41,42,43].

MAPKs transduce signals into the cell through a three-tiered cascade, from MAP3Ks (such as Raf1, Cot1, MTK/DLK or ASK1/TAK1/PTK1) through MAP2Ks (such as MEK1/2, MEK5, MKK7/MEK4 or MEK3/6) to MAPKs (ERK1/2, ERK5, JNKs, p38 kinases). Terminally, MAPKs activate several TFs (like c-Fos, c-Jun, PU.1, MEF2, ATF2, c-Myc and Sp1), activating genes responsible for proliferation, differentiation and cell death.

#### 2.1.1. MEK1/2-ERK1/2 Pathway

The ERK1/2 cascade is activated by several reactions initiated by extracellular signals and transmitted by growth factor receptors and cytokine receptors to the small G-coupled protein Ras1 [44], which can sequentially activate Raf1, MEK1/2 and, then, ERK1/2 kinases (Figure 1). When activated by MEK 1/2 phosphorylation, ERK1 and ERK2 are translocated to the nucleus and, in turn, phosphorylate transcription factors crucial for myeloid differentiation, such as C/EBPα, C/EBPβ or PU.1 [45,46,47]. Kinase suppressor of Ras 1 and 2 (KSR1 and KSR2), considered to be scaffold proteins that bring Ras1, Raf1 and MEK1/2 together, facilitate signaling through pathways mediated by ERK1/2 [48,49,50,51]. ERK1/2 have been shown to phosphorylate several different substrates, including ribosomal S6 kinase p90^RSK^ [52]. The Ras1-Raf1-MEK1/2-ERK1/2 pathway is an important positive regulator of monocytic and granulocytic differentiation [38,53,54]. 

#### 2.1.2. JNKs Pathway

The JNKs family is made up of three members: JNK1, JNK2 and JNK3 [55,56,57]. These kinases are activated by external stress, apoptotic stimuli and cytokines and are also known as SAPK (stress-activated protein kinases). These signals lead to their phosphorylation by upstream kinases (MEK4, MKK7) [58]. Although JNK1 and JNK2 have somewhat different actions on AML, in general, JNKs phosphorylate TFs, such as c-Jun, ATF-2, p53 and Elk-1, which, in turn, regulate the expression of specific genes to mediate cell proliferation, differentiation or apoptosis [59,60] (Figure 1). C-Jun is essential for monocytic differentiation of human AML cells, as a part of the AP1 TF [53,61].

#### 2.1.3. p38 Kinases Pathway

The p38 MAPKs are activated in cells by environmental stresses and pro-inflammatory cytokines, less often by growth factors. There are four members of the p38 MAPKs family, α, β, γ and δ, which display tissue-specific patterns of expression [62]. p38α and β, the “classical” isoforms, are ubiquitously expressed among tissues, whereas the expression of p38γ and δ appears to be more tissue restricted and, in 1,25D-treated AML cells, have positive effects of differentiation, unlike the classical isoforms [41,63,64,65,66]. The p38 kinases share about 40% sequence identity with other MAPKs, but they share only about 60% identity among themselves, which suggests highly diverse functions [67,68,69]. The p38 MAPKs are activated by phosphorylation by upstream kinases MKK3 and MKK6, although MKK4, the main activator of JNKs, has also been shown to activate p38 MAPKs [70]. Upon activation, p38 proteins translocate from the cytosol to the nucleus, where they orchestrate cellular responses by mediating phosphorylation of downstream targets that regulate apoptosis, cell cycle arrest, cell growth inhibition and differentiation [41,71,72] (Figure 1). Besides transcription factors, p38 kinases downstream targets are other kinases, such as MAPKAPK3 or MAPKAPK5 [65,73].

#### 2.1.4. MEK5-ERK5-MEF2C Pathway

Like the other branches of the MAPK family, the MEK5/ERK5 pathway has been implicated in cell survival, anti-apoptotic signaling, angiogenesis, cell motility, proliferation and cell differentiation [74]. However, ERK5 signaling can have both overlapping and distinct effects from the other MAPKs [75].

The principal activator of ERK5 is MEK5, which can be activated by MAP2Ks, such as MEKK2 and MEKK3 [76,77]. It has been shown that MEKK3 induces activation of the MEK5/ERK5 pathways through growth factor-induced cellular stimulation and oxidative stress [76,78] (Figure 1). ERK5 is activated by two phosphorylations: first, on the N-terminal TEY sequence, usually by MEK5 [79], and then by autophosphorylation on the ERK5 C-terminal transcriptional activation domain [80,81], which allows it to be translocated into the nucleus and to activate several TFs, including MEF2, Sap1, c-Fos and c-Myc [79,82,83,84]. The auto-phosphorylation of the ERK5 C-terminus may also be required for transcriptional activation [85]. The Cot1 oncogene can activate ERK5 [86,87] and can repress KSR1/2 [42,87]. 

### 2.2. PI3 Kinase-Akt1-mTOR Signaling

**Figure 2:**
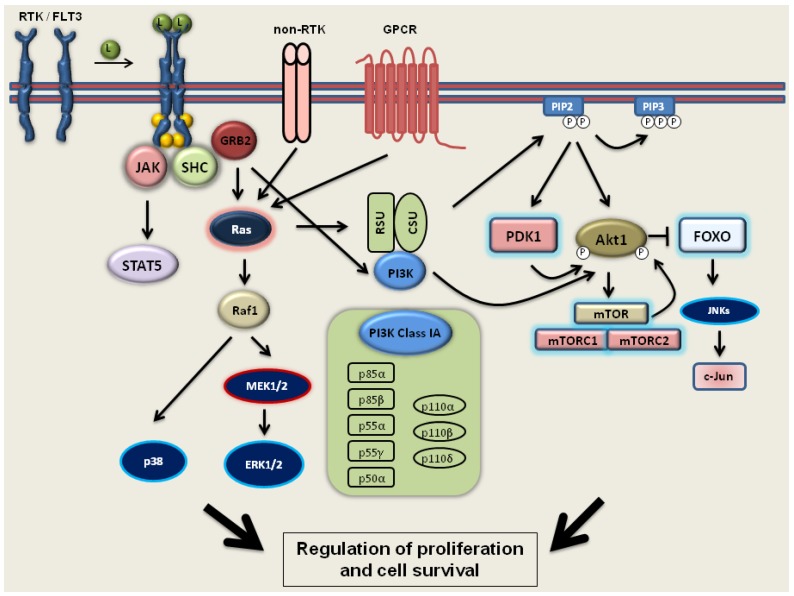
FLT3/PI3 kinase/Akt1/mTOR signaling pathways.

PI3Ks control the growth, motility, survival and differentiation of many normal and cancer cells [88,89,90]. The PI3Ks family is composed of heterodimeric proteins grouped into three main classes: I (IA and IB), II and III. Class IA enzymes are composed of three distinct catalytic subunits (p110α, p110β or p110δ), which associate with one of the regulatory subunits (p85α, p85β, p55α, p55γ or p50α) [91,92]. Class IB enzymes encompass one catalytic subunits (p110 γ) and two regulatory subunits (p101 or p87) [93]. Notably, p110α and p110β are ubiquitously expressed in most types of cells, whilst p110δ and p110γ are exclusively expressed in hematopoietic cells [92,94,95]. RTKs, non-RTKs, GPCRs and Ras1 are direct activators of class IA PI3Ks, whereas class IB enzymes are activated only by GPCRs and Ras1 [96,97]. Upon activation, the regulatory subunit mediates binding to the receptor, whereas the catalytic subunit phosphorylates PIP_2_ to yield PIP_3_. PIP_3_ initiates downstream signaling, such as the PDK1, Akt1, mTOR or FOXO family of TFs [98,99,100,101] (Figure 2). Direct constitutive activation of PI3K/Akt1/mTOR signaling occurs in the majority of leukemias, such as AML and ALL, Hodgkin’s lymphoma, lymphoproliferative disorders or myeloproliferative neoplasms [102,103,104].

After ligand (L) binding, FLT3 is phosphorylated (yellow circles), at TKD, and activates downstream pathways, such as PI3K/Akt1, MAPKs (MEK1/2, ERK 1/2) and STAT5. Two major classes of activating FLT3 mutations have been identified in AML patients: ITD and TKD point mutations. Mutations cause constitutive activation of FLT3 and aberrant activation of downstream signaling pathways and factor-independent growth. PI3K is activated downstream of RTKs, non-RTKs or GPCRs. Ras1 is a direct activator of class IA of PI3Ks, upon activation regulatory subunit (RSU) mediating the binding to the receptor, whereas the catalytic subunit (CSU) phosphorylates PIP_2_ to PIP_3_. PIP_3_ initiates downstream signaling, PDK1, Akt1, mTOR or FOXO.

### 2.3. FLT3 Signaling

FLT3 is encoded by a gene located on chromosome 13 and plays an important role in early hematopoiesis and development of myeloid precursors [105,106]. This transmembrane kinase belongs to the class III receptor tyrosine kinase family and is the most commonly mutated in AML [107,108,109]. The oncogenic mutations in FLT3 (ITD, internal tandem duplication in the juxtamembrane region or point mutation in the catalytic domain) cause ligand-independent dimerization of the FLT3 and its constitutive activation. Thus, the mutated FLT3 receptor activates downstream signaling pathways, such as PI3K, ERK1/2 and p38, LYN and STAT5 kinases, leading to the cytokine-independent proliferation (Figure 2) [109,110,111,112].

### 2.4. C/EBPα Signaling

The C/EBPα belongs to the family of basic leucine zipper TFs, which participate in the differentiation of several cell types, including myeloid progenitor cells from multipotent precursors [113,114,115]. There are two distinct isoforms of C/EBPα protein, full-length p42 and truncated p30, which lacks two N-terminal trans-activation domains [116]. Only the p42 isoform of C/EBPα can inhibit cell proliferation, while the p30 isoform can support the formation of granulocyte-macrophage progenitors in mice and can lead to the development of AML in the absence of p42 [117]. The relative levels of p42 and p30 in the cell can be regulated through mTOR signaling to control the transition of cell fate [118]. A genetic knockout of C/EBPα results in a complete block in the transition from common myeloid progenitors to the granulocyte/monocyte progenitor stage of differentiation [119]. Mutations in CEBPα occur in approximately 5% to 10% of *de novo* AML and is most common in cytogenetically normal AMLs [114,120]. 

### 2.5. Targeting by MicroRNAs

MicroRNAs are small, noncoding and highly conserved RNA molecules that regulate the expression of genes post-transcriptionally by binding to the 3′-UTR regions of the mRNA [121,122,123]. Several microRNAs are widely expressed in hematopoietic cells (*i.e.*, 106a, 128a, 146, 150, 155, 181a, 221, 222, 223), and their altered expression (e.g., by chromosomal translocations) has been correlated with leukemia [124]. Several studies have shown that specific patterns of microRNA expression are closely associated with cytogenetic and risk/survival predictions in AML patients [125,126,127,128]. Importantly, integration of microRNA and mRNA patterns of regulation can reveal the extent of co-regulation, which permits exquisite control of gene expression at the mRNA level [129,130].

### 2.6. Global Effects of VDDs on AML Cells

Most studies of signaling by VDDs were based on the examination of the expression of a single or a small number of genes. However, powerful new technology is evolving, which, combined with bioinformatics, is poised to transform this field. Therefore, the question can soon be answered of how the perturbations of cellular homeostasis by 1,25D or other VDDs influence the global gene expression. Interesting examples of this approach have recently been published by the Carsten group, which include a genome-wide analysis of VDR binding sites in THP-1 human monocytic leukemia cells. They identified by ChIP-seq 2340 VDR binding locations, of which 1171 occurred uniquely following short exposure to 1,25D and 520 without exposure to 1,25D [131]. Interestingly, it was found that 1,25D binding shifts the locations of VDR occupation to DR3-type response elements that surround its target genes, and there was a large variety of regulatory constellations of VDR binding sites. It is also becoming increasingly clear that VDR binding choices are highly specific for the cell type [130,131,132]. The biological significance may be derived from microarray analyses following 1,25D treatment, such as that which found that the monocytic marker CD14 and cathelicidin anti-microbial peptide were by far the most markedly upregulated genes in this scenario [131]. Among the genes upregulated early, as shown by the microarray analysis, the monocyte-specific genes and metabolism-related genes are two noticeable groups [132]. The effects of longer exposure to 1,25D include the finding that VDR binding sites are significantly enriched near autoimmune and cancer-associated genes identified from GWA studies [133]. Thus, GWA surveys can lead to deeper understanding of signaling by VDDs.

## 3. 1,25D as an Important Modifier of Signaling Pathways Disturbed in AML

1,25D is the physiological form of vitamin D that belongs to the family of secosteroid hormones [134,135]. Although the primary function of 1,25D is to maintain calcium and phosphorus metabolism [136], 1,25D is capable of inducing differentiation and inhibiting the proliferation of several types of normal and cancer cells, AML cells among them [137,138,139]. Exposure of AML cells to 1,25D results in a monocyte-like phenotype, which, upon prolonged exposure to 1,25D, becomes a macrophage-like phenotype, manifested by functional changes (phagocytosis accompanied by monocyte-specific esterase activity and the generation of reactive oxygen species). The phenotypic changes include altered morphology [140,141] and the expression of a receptor for complexes of lipopolysaccharides, CD14, and the adherence protein encoding the subunit of α_M_β_2_-integrin, CD11b [142,143,144,145]. 

There are several phases of 1,25D-induced differentiation of AML cells. In the initial phase, the cells continue normal proliferation and cell cycle progression. During this phase, high levels of MEK1/2, ERK1/2, JNKs and p38 kinases are essential [53,146,147]. Latter phases lead to the cell cycle block at the G1/S phase due to the elevated expression of CDK inhibitors, such as p21^Cip1/Waf1^ and p27^Kip1^ [148,149,150], and anti-apoptotic proteins, including Bcl-XL and Mcl1, which facilitate differentiation by increasing cell survival [151,152]. 

### 3.1. Activation of MAPKs by 1,25D

#### 3.1.1. Ras1-Raf1-MEK1/2-ERK1/2

The ERK1/2 signaling pathway maintains cell proliferation during the early stages of 1,25D-induced differentiation of AML cells (24–48 h). ERK1/2 are expressed at a high level and are activated by phosphorylation [53,146]. PD98059, the specific inhibitor of MEK1/2 [153], partially inhibits 1,25D-induced monocytic differentiation of HL60 cells. At the later phase, a high level of phosphorylated ERK1/2 decreases to the basal level, and then, ribosomal S6 kinase p90^RSK^ is activated [53,146,147]. This kinase, in turn, can activate C/EBPβ, the master TF for monocyte/macrophage differentiation [154,155]. C/EBPβ, which can be activated by phosphorylation by ERK1/2 [156], by p90^RSK^ [157] or by ERK5 [141] and can directly interact with the promoter region of CD14, activates its expression [45,156,158].

**Figure 3:**
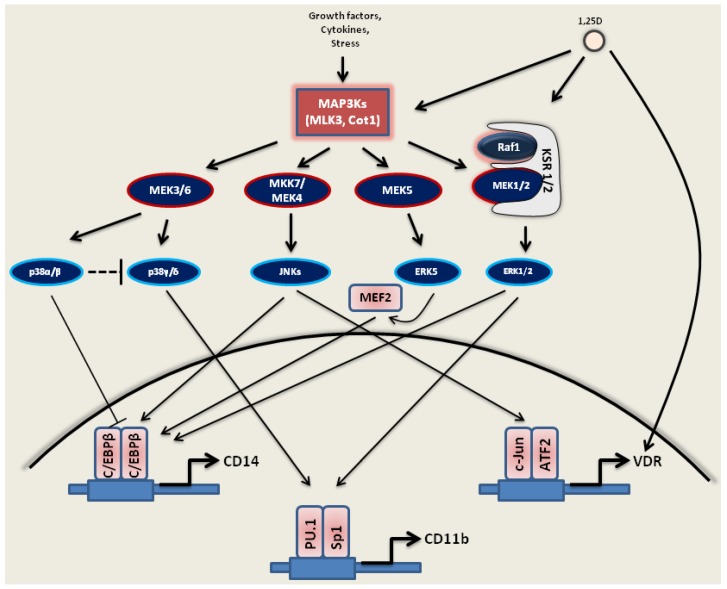
1,25D’s influence on MAPK signaling.

Raf1 signaling is a requisite for the latter stages of 1,25D-induced differentiation of HL60 cells. Raf1 mediates activation of p90^RSK^, but independently of the MEK1/2-ERK1/2 module [45]. Moreover, a platform for Raf1 phosphorylation, KSR1 and KSR2 [159] are also upregulated by 1,25D, augmenting the strength of the signal transmitted through Raf1 to downstream targets [49,160,161] (Figure 3). KSR2 knockdown decreases cell survival, which is accompanied by reduced Bcl-2/Bax and Bcl-2/Bad ratios and increased caspase-3 activating cleavage [162].

1,25D activates several MAPKs, such as MLK3, Cot1 and MEK1/2. The ERK1/2, ERK5 and JNK (JNK1/2) pathways have positive effects on monocytic differentiation, while the p38 MAPKs pathway may have a dual effect on differentiation. p38α and p38β have an inhibitory effect on monocytic, but not granulocytic differentiation of HL60 cells, while p38γ and δ may positively modulate monocytic differentiation of these cells. 1,25D can amplify the Raf1-MEK1/2-ERK1/2 pathway by direct transcriptional upregulation of KSR1 and KSR2, which act as scaffolds that coordinate signaling along the Ras1/ERK1/2 signaling. Also shown is the potential role of the C/EBPβ, c-Jun/ATF2 and PU.1/Sp1 TFs, which act as positive effectors of 1,25D signals by upregulating the expression of VDR, CD14 and CD11b.

#### 3.1.2. JNKs

During 1,25D-induced differentiation of AML cells, the expression level of JNK1 is highly elevated [147]. JNK1 activates c-Jun and ATF2, two major components of the AP-1 TF complex, as well as C/EBPβ and Jun B [61]. Moreover, the expression level of c-Jun is also elevated in those cells [163], which enhances the differentiation process [164]. Inhibition of JNK1/2 by the specific inhibitor, SP600125 [165], leads to the reduction of c-Jun and ATF2 phosphorylation, as well as to the decreased expression of Egr-1 and c-Fos, which results in the differentiation block [147,166,167]. Importantly, in AML cells resistant to 1,25D, JNK2 antagonizes JNK1 and is considered, at least in part, a negative regulator of the cell proliferation and resistance of those cells [168,169] (Figure 3).

#### 3.1.3. MAPK/p38 Kinases

The p38 kinases are also essential for 1,25D-induced differentiation of AML cells. It was shown that some functions generally attributed to p38 kinases, such as inhibition of 1,25D-induced differentiation [66], are only performed by the classical forms (p38α and p38β), as these, unlike p38γ and δ, are inhibited by SB203580, SB202190 and related compounds [66,170,171,172,173]. Because they exert a negative feedback upstream of p38α and p38, the isoforms p38γ and p38δ actually have a positive effect on 1,25D-induced differentiation of human AML cells [66] (Figure 3). Moreover, the inhibition of p38α and p38β leads to an upregulated expression of isoforms p38γ and p38δ in 1,25D-treated AML cell lines and in primary cultures [66].

#### 3.1.4. MEK5-ERK5-MEF2C

1,25D and its analogs upregulate the expression of ERK5, which positively regulates the early-stage monocytic differentiation of AML cells [75,87,141]. The pharmacological inhibitor of MEK5 (an upstream activator of ERK5), BIX02189 [174] and the inhibitor of ERK5 autophosphorylation, XMD8-92 [175], lead to the reduction of cell surface CD14 and an increase in CD11b expression [141]. ERK5 is a positive regulator of C/EBPβ TF, the direct activator of CD14, but negatively regulates the expression of C/EBPα [141]. MEF2C, a known downstream target of ERK5 [82,141], has recently been shown to be involved in 1,25D-induced AML differentiation and is reported to lie upstream of C/EBPβ and to control the expression of CD14, but not CD11b [176]. Importantly, the enzyme activity of Cot1, an upstream regulator of MEK5-ERK5-MEF2C, increases in AML cells during 1,25D-induced differentiation, as does phosphorylation of MEF2C, a downstream target of ERK5 [42,176] (Figure 3). It is also relevant that FLT3 kinase may activate MEK5 by its phosphorylation, which results in the activation of ERK5 of AML cells that have an internal tandem duplication in FLT3 [177]. As CD11b expression generally suggests terminal differentiation, the dissociation of CD14 and CD11b expression by the MEK5-ERK5-MEF2C signaling cascade implies that the monocytic characteristics of AML cells in the early phase of 1,25D-induced differentiation may just be an associated phenomenon, but is not a necessary component of any potential anti-cancer effect of 1,25D. 

### 3.2. The Effect of 1,25D on the PI3 Kinase-Akt1-mTOR Pathway

Activation of the PI3K/Akt1/mTOR pathway is important for 1,25D-mediated protection against apoptosis, as well as for the induction of the differentiation of AML cells [150,178,179,180,181]. Inhibition of PI3K by LY294002 or by Wortmannin accentuates the 1,25D-induced G1 to S phase cell cycle block in HL60 cells and is associated with an increased expression of p27^Kip1^ protein [150]. Moreover, LY294002 inhibits nuclear translocation of VDR and prevents activation of 1,25D target genes triggering monocytic differentiation [173].

### 3.3. The Influence of 1,25D on AML Cells with Mutated FLT3 Kinase

Only a few reports focus on the susceptibility of AML cells to the combined effects of 1,25D-induced differentiation and FLT3 kinase activating mutations. Studies performed on blast cells isolated from the peripheral blood of patients with diagnosed FLT3 mutations revealed that those cells exhibit resistance to 1,25D and to its “semi-selective” analogs [145,182]. This notwithstanding, AML cell lines with mutated FLT3 kinase, such as MV4-11 or MOLM-13, do respond to 1,25D-induced differentiation [145,183]. Treatment of elderly relapsed AML patients with cytotoxic agents, 1,25D and the FLT3 kinase inhibitor, CEP-701, gave highly variable results [13]. 

### 3.4. Effects of 1,25D on C/EBPα

It is well documented that C/EBPα is indispensable for granulocytes to develop, while C/EBPβ regulates the differentiation of monocytic cells [115,184,185]. In HL60 cells exposed to 1,25D, C/EBPα isoforms are transiently upregulated in the early stages (up to 24 h) of the differentiation process, while C/EBPβ isoforms are upregulated in a sustained fashion and parallel to the expression of CD14 and CD11b surface markers [45]. A generally accepted scheme assumes that 1,25D-induced expression of C/EBPβ allows the cells to bypass the granulocytic differentiation block caused by dysregulation of C/EBPα and switches the cells into monocytes [45,156,186].

### 3.5. The Effect of 1,25D on MicroRNAs

Relatively little is known regarding the influence of 1,25D on microRNA expression in AML cells, but it is postulated that similarly to the other types of cancer, the microRNA expression profile (“signature”) may be helpful for AML diagnosis and for the selection of suitable therapy. It was shown that during 1,25D-induced differentiation of AML cells, microRNA-181a and microRNA-181b are downregulated [187]. MicroRNA-181a inhibition by 1,25D results in an increase of p27^Kip1^ mRNA and protein level, which, in turn, leads to G1/S blockade [187,188,189]. Furthermore, microRNA-302c and microRNA-520c are downregulated by 1,25D in Kasumi-1 and K562 AML cell lines, where they enhance the susceptibility of those cells to natural killer cell-mediated cytotoxicity [190]. Other microRNAs downregulated by 1,25D in AML cells are microRNA-17-5p/20a/106a, microRNA-125b and microRNA-155, which target AML1, VDR and C/EBPβ [191].

**Figure 4:**
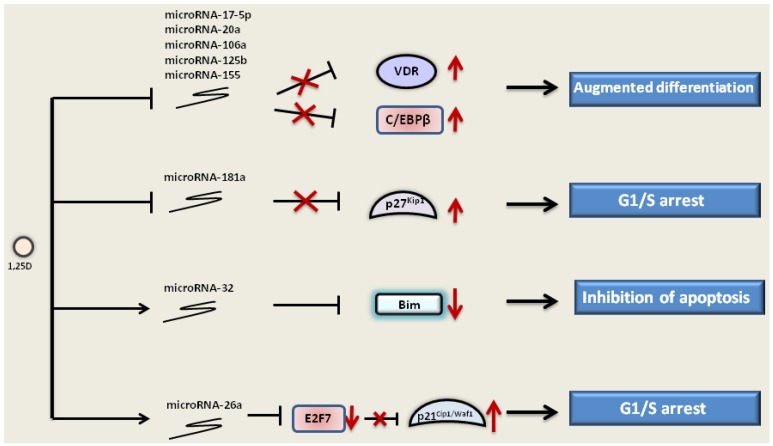
Regulation of microRNA expression by 1,25D in AML cells.

1,25D can upregulate microRNA-32, which targets pro-apoptotic protein Bim [192]. Decreased expression of microRNA-32 can sensitize AML cells to the cytotoxic agents, for instance arabinocytosine [192]. Another microRNA upregulated by 1,25D in AML cells is microRNA-26a [193], which targets transcriptional repressor E2F7 [194]. The repression of E2F7 by miR-26a contributes to the increased expression of p21^Cip1/Waf1^ observed during 1,25D-induced monocytic differentiation of AML cells. Moreover, silencing of E2F7 results in inhibition of c-Myc activity and downregulation of its transcriptional target, the oncogenic miR-17-92 cluster [194] (Figure 4).

1,25D down-regulates (⟞) the expression of microRNA-181a, which is a negative regulator of p27^Kip1^. This causes the block of the negative action of microRNA-181a (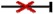) and elevated expression of p27^Kip1^ (↑). 1,25D also downregulates other microRNAs, such as microRNA-106a, -20a and -155, that target and inhibit the expression of VDR and C/EBPβ. 1,25D can up-regulate (→) microRNA-32, which targets pro-apoptotic protein Bim and inhibits its expression (↓). Furthermore, microRNA-26a is upregulated by 1,25D. MicroRNA-26a inhibits transcriptional repressor E2F7, which, in turn, no longer inhibits p21^Cip1/Waf1^, and its expression is elevated. Regulation of microRNA expression by 1,25D leads to the augmented differentiation, inhibition of apoptosis and cell cycle arrest in the G1/S phase. 

## 4. Potentiators of 1,25D-Induced Differentiation of AML Cells

1,25D-induced differentiation of AML cells may be augmented by several natural and chemical compounds (Table 1). One such compound is carnosic acid (CA), the plant-derived polyphenolic antioxidant. CA alone is weakly cytostatic to AML cells, but in combination with 1,25D, increases differentiation and upregulates the expression of ERK5, c-Jun and AP1 [87,166,169,195,196,197]. Moreover, CA together with a 1,25D analog, doxercalciferol, decreases the expression level of microRNA-181a [189]. Similarly to CA, other plant antioxidants, curcumin and silibinin, can inhibit AML cell growth when used alone, but show synergistic or additive effects on differentiation when combined with VDDs [195,198,199,200]. 

It was also shown that an inhibitor of the Akt1/mTOR pathway, RAD001 (Everolimus), potentiates 1,25D-induced growth arrest and differentiation of AML cells, due to the enhancement of 1,25D-mediated transcriptional activation of p21^Cip1/Waf1^ in association with increased level of the acetylated forms of histone H3 and VDR bound to the p21^Cip1/Waf1^ promoter [201].

A large number of compounds can potentiate 1,25D-induced differentiation of AML cells [198,202]. Natural compounds include plant polyphenols, carnosic acid, curcumin or silibinin [195,198,203]. Other compounds, such as iron chelators [204] or chemical inhibitors [205], are also capable of enhancing 1,25D action. 

**Table 1:** Examples of potentiators of 1,25D-induced differentiation of AML cells.

Compound	Characteristic	Mode of Action with 1,25D	Citations
*Nutlin 3a*	*cis*-imidazoline analog, inhibits interaction between Mdm2 and p53	▪ downregulation of Bcl-2, MDMX, KSR2, phospho-ERK2 ▪ upregulation of PIG-6	[152]
*Carnosic acid*	natural benzenediol abietane diterpene from rosemary	▪ upregulation of ERK5, c-Jun and AP1 ▪ downregulation of microRNA-181a expression	[189,196,197]
*Curcumin*	diarylheptanoid, natural phenol from turmeric	▪ activation of caspase-3, -8 and -9	[195]
*Silibinin*	flavonolignan from the milk thistle seeds	▪ upregulation of c-Jun and C/EBPβ	[199,200]
*Everolimus*	40-*O*-(2-hydroxyethyl) derivative of sirolimus	▪ inhibition of Akt/mTOR	[201]
*Deferasirox*	iron chelator	▪ induction of VDR expression and phosphorylation	[204,207]
*Q-VD-OPh*	pan-caspase inhibitor	▪ upregulation of HPK1 and c-Jun	[205]
*Indomethacin*	non-steroid inhibitor of cyclooxygenase	▪ inhibition of phospho-Raf1	[206]

Other compounds that can enhance 1,25D-induced differentiation of AML cells are COX1 and COX2 inhibitors [206]. It was found that a combination treatment with 1,25D and non-specific COX inhibitors acetyl salicylic acid (ASA) or indomethacin can robustly potentiate the differentiation of several AML cell lines and that ASA ± 1,25D is effective in enhanced differentiation of primary AML cultures. Increased cell differentiation is paralleled by arrest of the cells in the G1 phase of the cell cycle and by increased phosphorylation of Raf1 and p90^RSK1^ proteins [206].

Iron chelating agents, such as deferasirox, also turned out to be an effective enhancer of 1,25D-induced differentiation of AML cells [204]. This compound induces expression and phosphorylation of the VDR. The combination of iron-chelating agents and 1,25D resulted in the reversal of pancytopenia and in blast differentiation, suggesting that iron availability modulates myeloid cell commitment and that targeting this cellular differentiation pathway together with the conventional differentiating agents can provide a therapeutic benefit for an AML patient [204]. This conclusion is reinforced by the subsequent retrospective study, which showed that the combination of deferasirox and vitamin D improves overall survival in elderly patients with AML after demethylating agent failure [207]. In accordance with these feasibility studies, a phase 1 and 2 clinical trial (NCT01718366) of combined deferasirox, vitamin D, and azacitidine in high risk MDS is in progress.

## 5. Clinical Trials with VDDs Targeting Signaling Pathways in AML

Poor responsiveness to standard chemotherapy is still a problem for a significant number of patients with neoplastic diseases. While the current focus in the field is on individualized therapy based on molecular features of the disease, the great heterogeneity of mutations in AML makes this a remote aim. Thus, the possibility that a differentiation-based approach can be used for a large subset of AML patients has been attractive. However, the attempts to utilize the differentiation properties of VDDs have had so far minimal success, possibly due, at least in part, to the variable levels of vitamin D receptors in the malignant cells [18]. 

A recent review by Kim *et al*. [15] includes a list of clinical trials mainly conducted in the early 1990s, which seem to have mostly “fizzled out”, that have not led to any major advances in the treatment of AML. Harrison and Bershadskiy [16] describe these clinical trials in depth and list two more trials in patients with MDS, often a pre-leukemic disease, but neither trial led to dramatic or promising results. More recently, several other trials of VDDs have been conducted in MDS patients; however, the results of those have not yet appeared in the literature, and the only phase 3 trial that could be found at this time (NCT00804050) has been terminated, reportedly due to “difficulties in enrollment”. Thus, there remains substantial uncertainty as to whether VDDs, with or without potentiators that were used in the majority of experiments reported to date, will have a significant therapeutic effect in AML. It would appear that at least part of the problem is that the potentiators/enhancers so far used with VDDs had no clearly defined mechanism of potentiation of their combined actions. These have included new cytotoxic agents and their combinations with cell cycle, histone deacetylase inhibitors, monoclonal antibodies, FLT3 kinase inhibitors and hypomethylating agents currently used as enhancers of cytotoxic therapy (Table 2). Such agents do have a clearly defined basis of action as single agents, but the rationales of the potentiation of the differentiation agents are not clear. Most relevant to this topic are phase I/II trials of MEK inhibitors AS703026 3 (pimasertib) and GSK1120212 (trametinib). These trials investigate the safety, pharmacokinetics, pharmacodynamics and clinical activity of these compounds in AML patients with all subtypes, except FAB M3 [208,209,210]. Further problems in drawing conclusions regarding the efficacy of VDDs in AML based on the clinical trials reported to date are the great heterogeneity of the patient populations studied and the variability in the dose and schedule of the VDDs used to date.

It is proposed that a better understanding of the signaling pathways underlying VDD actions may stimulate the generation of new concepts for clinical trials of VDDs with potentiators. Perhaps a simultaneous, or sequential, targeting of pathways described here by VDDs and enhancers or inhibitors of these pathways will provide conceptually new regimens for clinical trials of VDDs. The importance of optimal sequencing in differentiation therapy is suggested by a recent report that survival of patients with AML/MDS was improved by agents, which included VDDs, administered during the maintenance of remission induced by chemotherapy [211]. Furthermore, in addition to pathway inhibitors, pathway activators should also be considered for the enhancement of VDD therapeutic activity.

**Table 2:** New agents in AML clinical trials. The recent clinical trials of AML have focused on new cytotoxic drugs, cell cycle and histone deacetylase inhibitors, monoclonal antibodies, FLT3 and MEK kinase inhibitors or hypomethylating agents. These were conducted without VDDs.

Target	Compounds	Phase	Status of the study	Examples of studies
Cell cycle inhibition	rigosertib	I/II	ongoing, recruitment closed	NCT01167166
	volasertib	I	ongoing, recruitment opened	NCT02003573
Cytotoxicty	clofarabine	I	ongoing, recruitment opened	NCT01289457
	sapacitabine	III	ongoing, recruitment opened	NCT01303796
	vosaroxin	I/II	ongoing, recruitment opened	NCT01893320
DNA hypomethylation	azacitidine	II	ongoing, no recruitment	NCT01358734
	decitabine	II	ongoing, recruitment opened	NCT02188706
	SGI-110	II	ongoing, recruitment opened	NCT02096055
FLT3 small-molecule inhibitors	crenolanib	II	ongoing, recur	NCT01657682
	midostaurin	I/II	itment opened	NCT01093573
	sorafenib	II	ongoing, recruitment opened	NCT02196857
			not yet open for recruitment	
Histone deacetylase inhibitors	panobinostat	I/II	ongoing, recruitment opened	NCT01451268
	pracinostat	II	ongoing, recruitment opened	NCT01912274
	vorinostat	I	ongoing, no recruiment	NCT00875745
Monoclonal antibodies	gemtuzumab ozogamicin	III	ongoing, recruitment opened	NCT00893399
	SGN33a	I	ongoing, recruitment opened	NCT01902329
MEK inhibitors	MEK162	I/II	ongoing, recruitment opened	NCT02089230
	trametinib (GSK1120212)	II	ongoing, recruitment opened	NCT01907815

## 6. Conclusions and Perspectives

It is clear that despite the strong epidemiological evidence that optimal levels of vitamin D reduce overall mortality by at least 7% [212,213], while low vitamin D levels are associated with adverse outcomes in AML [214], translation of this knowledge to cancer prevention or treatment has been disappointingly slow. The early focus on the generation and testing of countless vitamin D analogs for cancer treatment has not led to encouraging results, and combinations of 1,25D or analogs with cytotoxic agents have not led to conclusive results in neoplastic diseases, including AML [215,216,217]. It appears more likely that the therapeutic regimens for AML will require the addition of small molecule inhibitors, or enhancers of signaling pathways, or entirely new strategies. The latter can capitalize on the known changes in gene expression elicited by 1,25D, summarized here. Although current excitement in the field of cancer therapy is largely directed to targeting specific mutations, found successful for CML and APL, the vast majority of leukemia cases have a highly heterogeneous set of mutations, making targeting not likely in the foreseeable future. Thus, randomized trials with patients with AML other than APL need to be organized, with time to relapse as the main end point, for confirming the findings that MAPKs, and other signaling cascades, present important auxiliary targets that can enhance the effectiveness of the cytotoxic therapy or immunotherapy of cancer.

## Abbreviations

1,25D: 1,25-dihydroxyvitamin D_3_
Akt1: protein kinase B
ALL: acute lymphoblastic leukemia
AML: acute myeloid leukemia
AP1: activator protein 1
APL: acute promyelocytic leukemia
ATF2: activating transcription factor 2
ATRA: all-trans retinoic acid
CA: carnosic acid
C/EBPs: CCAAT enhancer binding proteins
CML: chronic myelogenous leukemia
Cot1: cancer Osaka thyroid 1
COX: cyclooxygenase
ERK: extracellular signal-regulated protein kinase
FLT3: FMS-like tyrosine kinase 3
GPCRs: G-protein-coupled receptors
GWA: genome-wide association
JNKs: c-Jun N-terminal kinases
KSR: kinase suppressor of Ras
MAPK: mitogen-activated kinase
MAP2K: mitogen-activated kinase kinase
MAP3K: mitogen-activated kinase kinase kinase
MDS: myelodysplastic syndrome
MEF2: myogenic enhancer factor 2
mTOR: mammalian target of rapamycin
non-RTKs: non-receptor tyrosine kinases
PDK1: 3-phosphoinositide-dependent kinase 1
PI3K: phosphatidylinositol 3-linase
PIP_2_: phosphatidylinositol-4,5-bisphosphate
PIP_3_: phosphatidylinositol-3,4,5-trisphosphate
RTKs: receptor tyrosine kinases
TF: transcription factor
TKD: Tyrosine Kinase Domain
VDDs: vitamin D compounds/derivatives
VDR: vitamin D receptor
